# Is There an Association between Keloids and Blood Groups?

**DOI:** 10.5402/2012/750908

**Published:** 2012-12-06

**Authors:** Abas Mouhari-Toure, Bayaki Saka, Koussaké Kombaté, Sefako Akakpo, Palakiyem Egbohou, Kissem Tchangaï-Walla, Palokinam Pitche

**Affiliations:** ^1^Department of Dermatology, Teaching Hospital of Tokoin, University of Lomé, 08 BP 80598, Lomé, Togo; ^2^Department of Anesthesiology, Teaching Hospital of Tokoin, University of Lomé, 08 BP 80598, Lomé, Togo

## Abstract

*Objective*. The aim of the study is to investigate the possible associations between the blood groups ABO and Rhesus systems and the presence of keloids in patients with black skin. *Method*. This case-control study was conducted between September 2007 and August 2011 comparing dermatologic outpatients with keloids to matched controls recruited in preanesthetic consultation at Tokoin Teaching Hospital of Lomé (Togo). *Results*. The distribution of different ABO blood groups and Rhesus blood groups in both groups (cases versus controls) was not significantly different. This distribution of different blood groups was superimposed on the general population of blood donors at the National Blood Transfusion Center of Lomé. Univariate analysis between each blood group and the presence of keloid does not yield any statistically significant association between blood groups and presence of keloids in the subjects. *Conclusion*. The study shows no significant association between blood groups and the presence of keloids in our patients. Further investigation needs to be conducted to elucidate this hypothesis further by conducting multicenter studies of several ethnic groups.

## 1. Introduction

Keloids are defined as intradermal tumors corresponding to an abnormal response of tissue to injury in predisposed individuals [1]. Factors that play a major role in keloid development are genetic predisposition coupled with some forms of skin trauma. Transforming growth factor has been implicated as the main factor responsible for the abnormal proliferation of keloid fibroblasts and excessive production of collagen.

The red cell alloantigens of blood group are present on the membrane surface of red blood cells and certain epithelial cells [2]. Several publications have documented the associations between blood group and certain skin diseases [3–9].

In a study conducted in 1969 to 1970 on 486 patients with keloids in the city of Madras, South Indian, Ramakrishnan et al. in 1974 had found a predominance of blood group A, compared to the local population of this city (34.96% against 21.38% in the local population) [10]. And up to today, no studies have confirmed this association. In the context of our study, in the sub-Saharan Africa, the prevalence of keloids is high, and keloids represent 1.2% of dermatological consultations in Lomé [11]. In addition, the difficulties of management are arguments to search for possible factors associated with these conditions, and for their possible prevention. The main objective of this study is to investigate possible associations between blood groups ABO and Rhesus systems and the presence of keloids in patients with dark skin.

## 2. Method

This study is a case-control investigation conducted between September 2007 and August 2011. The participants consisted of patients (cases) who were recruited in three outpatient dermatology departments in Lomé in Togo. The controls were systematically recruited in the Anesthesiology Department of Teaching Hospital of Tokoin, during a preanesthetic consultation, ignoring the indication for surgery during the same period. The diagnosis of keloids was clinically made after an interview and physical examination by a dermatologist.

### 2.1. Inclusion Criteria for Cases

(i) Patients who consulted for keloid; (ii) patients who consulted for any other reason, but for whom physical examination revealed the presence of keloid on the skin.

### 2.2. Noninclusion Criteria for Cases

(i) Patients with only acne keloidalis nuchae; (ii) patients with skin phenotype I to V of the Fitzpatrick skin phototype classification.

### 2.3. Inclusion Criteria for Controls

Among the subjects enrolled in pre-anesthetic consultation at Tokoin Teaching Hospital of Lomé, the controls were consecutively recruited according to their ages and gender. Each case was matched with two controls on age (±3 years) and gender.

### 2.4. Noninclusion Criteria for Controls

After questioning and doing assessment of the entire integumentary system, any control subject with a suspicious hypertrophic scar or keloid was not included in the study.

### 2.5. Ethical Consideration

We obtained informed consent from each patient included in the study. Patients who did not sign the consent form were not included in the study.

### 2.6. Conduct of the Study

The clinical features of keloids were recorded in patients. Blood group ABO and Rhesus system were performed according to national guidelines: achieving the grouping by two technicians blinded by the two methods globular (Beth-Vincent test) and plasma (Simonin-Michon test) for the ABO system and using an anti-D serum test in the search of the Rhesus factor for the Rhesus system).

### 2.7. Statistical Analysis

The data collected were analyzed using Epi Info 3.3.2. Comparison of the distribution of blood groups was performed between the two groups using the Chi-square test (or Fisher exact test for frequencies less than 5). Associations between blood groups and keloids were evaluated by calculating the odds ratio in the univariate analyzes.

## 3. Results

A total of 82 patients with keloids were recruited and 164 controls matched to the cases. All these subjects were skin phototype VI (black subjects or Negroid), native to West Africa. The mean age (± SD) of the subjects was 31.3 ± 13 years with 52.4% of female subjects and 47.6% of male.

The 82 patients had an average of 4.6 keloids, and among them 76.8% had at least two keloid scars (Table 1). The keloids lasted for more than six months in 86.7% of patients. The mean size of keloids (long axis of the larger keloid) was 7 cm (range: 1 cm–40 cm). The keloids were predominantly located on the trunk (57.3%) and the upper limbs (34.1%).

Physical trauma was the major cause in 41 cases (50%), while the inflammatory lesions (acne, folliculitis, boils, chickenpox/herpes zoster) counted for 42.7% (35 cases). Keloids were considered as spontaneous in 11 cases (13.4%). Thirteen patients (15.9%) reported multiple causative lesions.

Distributions of different ABO blood groups (A, B, AB, O) in both groups (cases versus controls) were not significantly different (Chi-square = 1.89, degrees of freedom = 3, *P*  value = 0.5952). This distribution of different ABO blood group systems was superimposed on the general population of blood donors at the National Blood Transfusion Center of Lomé (NBTCL) (Table 2).

The proportion of Rhesus-positive subjects was not significantly different between cases (90.2%) and controls (93.3%) (Chi-square = 0.71, *P* = 0.3985). This distribution of Rhesus blood group system in both groups (cases versus controls) was superimposed on the general population of blood donors at the NBTCL (Table 2).

Univariate analysis between each blood group and the presence of keloid does not note a statistically significant association between blood groups and the presence of keloids in our subjects (Table 2).

## 4. Discussion

The main objective of this study is to compare the distribution of blood group between subjects with keloids and controls recruited in hospitals. No significant association has been found between blood groups and the presence of keloids in our patients. In India, Ramakrishnan et al. in 1974 had found that there were more subjects of blood group A among the patients with keloids, than in the general population [10]. It was a simple observation, and no comparison was performed to eliminate a possible coincidence. In this study, not only were the distributions of blood groups not different in the two groups, but also they were superimposable on the distribution of blood groups in the general population of blood donors at the NBTCL, which is substantially the same in sub-Saharan Africa [12, 13].

Several etiological factors have been proposed to explain the occurrence of keloids [1]. The factors that play a predominant role in the development of keloids are genetic predisposition associated with certain forms of skin trauma. The existence of familial cases of keloid disease, the onset of the disease in twins, and the high prevalence in some ethnic groups are strong arguments in favor of genetic predisposition for keloids [14, 15]. The mode of inheritance is autosomal dominant with incomplete penetrance in most studies in African-Americans and Asians [16–18]. The susceptibility gene of keloid was found on chromosome 2q23 in the Japanese families; and on chromosome 7p11 in the African-American families [16]. However, the genes encoding for blood group antigens are located on chromosome 9q34.2* (for the ABO system); and on chromosome 1p36.11 (for the Rhesus system) [19, 20]. Concerning the major histocompatibility complex, it was found an association between keloid and HLA-DRB1* 15 (among Caucasians), HLA-DQA1* and DQB1* (among Chinese) [21, 22]. The results of this study, and the arguments cited above suggest that the association between blood groups and keloids is an interesting hypothesis but very unlikely. Multicenter studies of several ethnic groups should help elucidate this hypothesis definitively.

## Figures and Tables

**Table 1:** Clinical features of keloids.

		Number	Percentage (%)
Number of keloids	One keloid	19	23,2
	2 to 5 keloids	43	52,4
	6 to 10 keloids	10	12,2
	More than 10 keloids	10	12,2

Size of keloids*	1 to 5 cm	48	58,5
	6 to 10 cm	17	20,7
	11 to 15 cm	11	13,4
	16 to 20 cm	3	3,7
	More than 20 cm	3	3,7

Duration of keloids	Less than 6 months	11	13,4
	6 to 12 months	18	22
	13 months to 5 years	18	22
	More than 5 years	35	42,7

Locations of the keloids^¶^	Head	19	23,2
	Neck	11	13,4
	Trunk^†^	47	57,3
	Upper limbs^‡^	28	34,1
	Lower limbs	9	11

Family history of keloids (cases/controls)	Ascendants	18/31	
	Progeny	2/0	
	Collateral	3/2	
	No or I don't know	59/131	

*The length of the major axis of the largest keloid of each patient.

^†^On the trunk: presternal region = 33 cases.

^‡^On the upper limbs: shoulders = 10 cases.

^¶^Some patients had multiple locations.

cm: centimeter.

**Table 2:** Calculation of odds ratio of potential risk factors.

	Cases *n* (%)	Controls *n* (%)	Odds ratio (CI)	NBTC-Lomé* (%)
Blood group A	19 (23,2)	39 (23,8)	OR = 0,97 (IC = 0,49–1,89)	21,1
Blood group B	22 (26,8)	49 (29,9)	OR = 0,86 (IC = 0,46–1,62)	25,6
Blood group AB	2 (2,4)	9 (5,5)	Fisher exact test^†^: *P* = 0.2282	4,7
Blood group O	39 (47,6)	67 (40,9)	OR = 1,31 (IC = 0,74–2,32)	48,6
Rhesus positive	74 (90,2)	153 (93,3)	OR = 0,67 (IC = 0,24–1,9)	91

*NBTC: National Blood Transfusion Centre (mean of years 2006, 2007, 2008).

^†^Fisher exact test used for frequencies less than 5.

OR: odds ratio.
